# Evaluation of Immediate Postpartum Long-Acting Reversible Contraception for Reducing Short-Interval Pregnancies

**DOI:** 10.7759/cureus.83395

**Published:** 2025-05-03

**Authors:** Andrew Grover, Amanda Ricard, Heather Cunningham, Molly Haas, Joanne N Quinones, Amanda Flicker

**Affiliations:** 1 Obstetrics and Gynecology, University of South Florida Morsani College of Medicine, Tampa, USA; 2 Obstetrics and Gynecology, Lehigh Valley Health Network, Allentown, USA; 3 Obstetrics and Gynecology, Thomas Jefferson University, Philadelphia, USA; 4 Maternal-Fetal Medicine, Lehigh Valley Health Network, Allentown, USA

**Keywords:** birth intervals, long-acting reversible contraception, postpartum contraception, pregnancy outcome, short interpregnancy interval

## Abstract

Background: Short interpregnancy intervals (conception occurring <18 months after delivery or pregnancy loss) are associated with increased risk of adverse outcomes such as preterm delivery, low birth weight, and invasive placental pathologies. To reduce unintended short-interval pregnancy (SIP) among people desiring contraception, our health network in 2019 began offering immediate postpartum long-acting reversible contraception (LARC), using copper and hormonal intrauterine devices (IUDs) and etonogestrel implants. This study evaluated the impact of the initiative on the incidence of SIP at our institution.

Methods: We conducted a retrospective cohort study of patients on government insurance who delivered between July 1, 2019, and March 31, 2020. The exposure of interest was LARC placement in the immediate postpartum period. The outcome of interest was confirmed pregnancy within 18 months of delivery. Exclusion criteria were immediate postpartum sterilization, cesarean or peripartum hysterectomy, or fetal demise. SIP rates were compared between those opting for LARC immediately after delivery (pp LARC group) and those who declined (no pp LARC group). A logistic regression model controlled for potential confounders.

Results: Of the 1,126 patients who met the inclusion criteria, there were 140 in the pp LARC group (68 etonogestrel implant, 54 levonorgestrel IUD, and 18 copper IUD) and 986 in the no pp LARC group. The pp LARC group experienced a lower proportion of SIP (7.9% versus 23.8%, P < 0.001). In adjusted analysis, immediate postpartum LARC reduced the rate of SIP (adjusted risk ratio (ARR): 0.28, 95% confidence interval (CI): 0.14-0.55, P < 0.001).

Conclusion: Providing LARC shortly after delivery shows promise for patient uptake and prevention of SIP among individuals wishing for contraception. Expanded studies are warranted.

## Introduction

Short-interval pregnancy (SIP) is defined as a pregnancy that occurs within 18 months of a previous delivery or pregnancy loss [1]. The American College of Obstetricians and Gynecologists (ACOG) advises avoiding interpregnancy intervals less than six months and also reports on the increased risks of adverse outcomes for subsequent pregnancies occurring between six and 18 months [1]. SIP has been associated with increased risk of gestational diabetes and placenta accreta spectrum [2-4]. In addition, SIP increases the risk of adverse neonatal outcomes, including preterm birth, low birth weight, and small size for gestational age [5]. According to the 2006-2010 National Survey of Family Growth, approximately 35% of pregnancies occurred within 18 months of a prior pregnancy [6], identifying a substantial area where risk can be reduced in individuals interested in preventing pregnancy.

Long-acting reversible contraceptive (LARC) methods, such as intrauterine devices (IUDs) and subdermal implants, offer solutions to the limitations of daily oral contraception by preventing pregnancy without active intervention from the individual. Despite these options, about 45% of US pregnancies in 2011 were unintentional [7].

While immediate postpartum LARC has been shown to be effective for mitigating unintended pregnancy, implementation of programs providing access to this contraceptive option has posed challenges. In April 2019, our health network began an initiative around this service. This study evaluates the usage of immediate postpartum LARC after the implementation of the program and the incidence of SIP during the first 18 months after delivery for patients within our population.

## Materials and methods

This retrospective cohort study included patients aged ≥18 on government insurance plans who delivered between July 1, 2019, and March 31, 2020, at one of two campuses of a southeastern Pennsylvania hospital network in the United States. The exposure of interest was the placement of a LARC method immediately after delivery and before discharge from the hospital after delivery. LARC options included levonorgestrel IUD, copper IUD, or etonogestrel implant. The outcome of interest was a documented pregnancy within 18 months after delivery. Data extracted for analysis from medical records included patient demographics, LARC option, and pregnancy status 18 months post-delivery. Patients were excluded if they underwent immediate postpartum sterilization, underwent cesarean or peripartum hysterectomy, or experienced a fetal demise during their index pregnancy.

Comparisons between groups (those who received immediate postpartum LARC (pp LARC group) and those who did not (no pp LARC group)) were made with Student's t-test or Wilcoxon rank-sum test for continuous variables and chi-square analysis or Fisher's exact test for categorical variables. Maternal age was evaluated as both a continuous variable and a categorical variable (maternal age 18-21 years versus >21 years of age). Logistic regression was used to identify independent predictors of SIP, controlling for potential confounders, and adjusted risk ratios (ARRs) with 95% confidence intervals (CIs) were derived from these models. The sample was powered at 90% to detect a 20% reduction in SIP rates, with statistical significance set at 0.05. Analysis of data was performed using Stata SE version 16 (StataCorp LLC, College Station, TX).

A sample size analysis revealed that to find a reduction in SIP rates from 35% to 15%, each comparison group needed 96 patients. This sample size provided a statistical power of 90%, and statistical significance was set at 0.05.

This study was approved by the health network's institutional review board.

## Results

**Figure 1:**
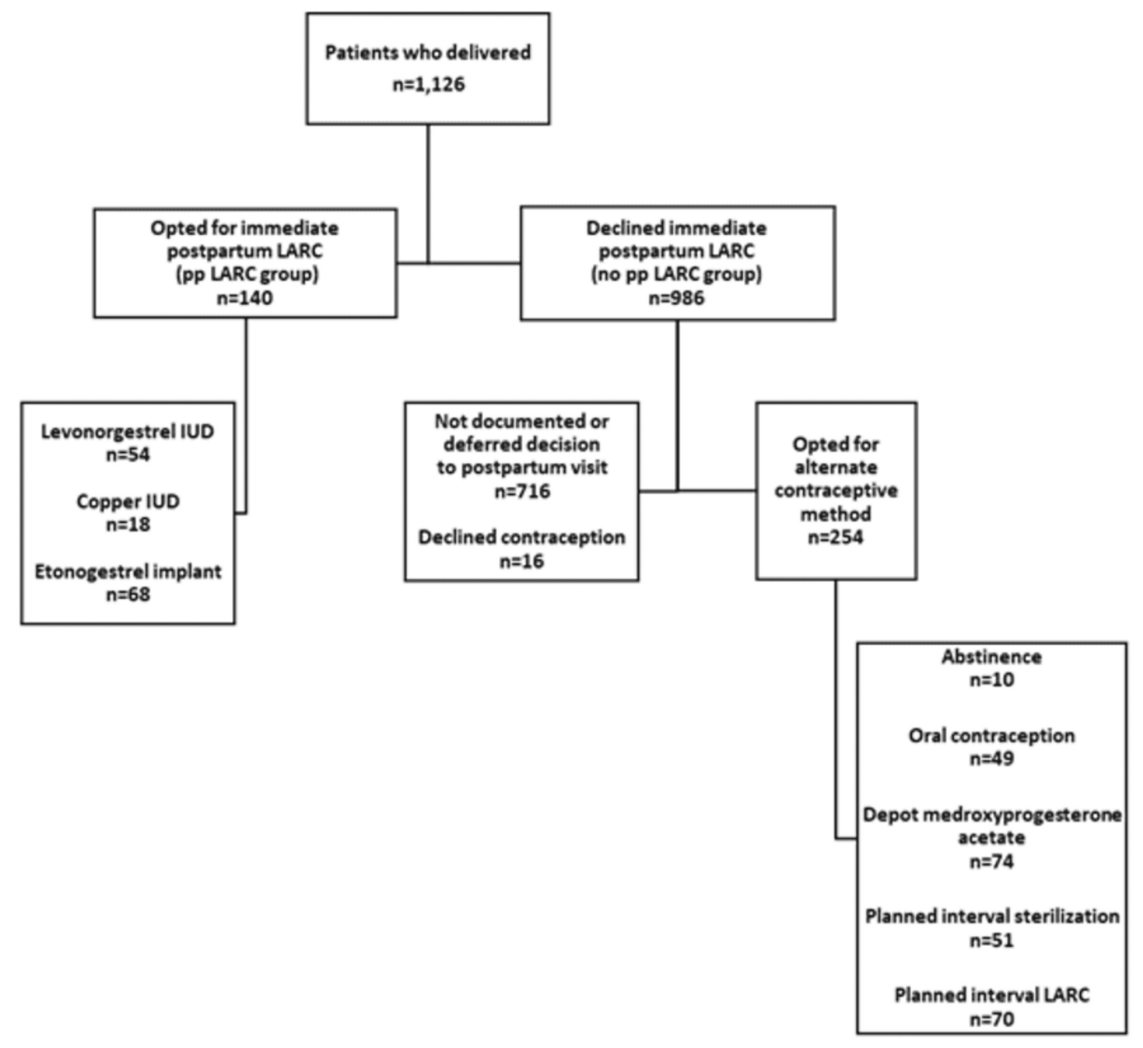
Flow diagram LARC: long-acting reversible contraception, pp LARC: postpartum long-acting reversible contraception, IUD: intrauterine device

Of the 1,126 patients who met the inclusion criteria, 140 (12.4%) were in the pp LARC group, and 986 (87.6%) were in the no pp LARC group (Figure 1). Of those who opted for immediate LARC, 68/140 (48.6%) received an etonogestrel implant, 54/140 (38.6%) levonorgestrel IUD, and 18/140 (12.9%) copper IUD. Patients who opted for LARC were younger than those who did not (mean age: 26.0 versus 27.4 years, P = 0.005), and more were non-White (61.4% versus 45.4%, P < 0.001) and Hispanic (69.8% versus 44.8%, P < 0.001) (Table 1).

**Table 1:** Demographic characteristics of patients who delivered between July 1, 2019, and March 31, 2020, by comparison group (N = 1,126) Note: Bold numbers indicate statistically significant P-values. pp LARC: postpartum long-acting reversible contraception, SD: standard deviation, BMI: body mass index

	No pp LARC group (n = 986)	pp LARC group (n = 140)	P-value	Statistical test utilized
Maternal age mean (SD)	27.4 (5.5)	26.0 (5.2)	0.005	Student's t
Maternal age 18-21 years (number (%))	139 (14.1)	39 (27.9)	<0.001	Chi-square
Parity median (range)	2 (1-3)	2 (1-3)	0.05	Wilcoxon rank-sum
Nulliparity(number (%))	385 (39.1)	40 (28.6)	0.02	Chi-square
Living children (number (range))	2 (1-3)	2 (1-3)	0.02	Wilcoxon rank-sum
Pregravid BMI (mean (SD))	28.5 (7.6)	27.7 (6.4)	0.23	Student's t
Race (number (%))				
Non-White	447 (45.4)	86 (61.4)	<0.001	Chi-square
White	538 (54.6)	54 (38.6)		
Ethnicity (number (%))				
Hispanic/Latino	433 (44.8)	97 (69.8)	<0.001	Chi-square
Marital status, married (number (%))	213 (23.6)	22 (16.4)	0.06	Chi-square

SIP occurred in 246 patients, 11 (7.9%) in the pp LARC group and 235 (23.8%) in the no pp LARC group (P < 0.001). A post hoc analysis excluding the 732 patients who declined contraception or deferred the decision to the postpartum visit found similar SIP rates (7.9% in the pp LARC group versus 22.1% in the no pp LARC group, P < 0.001).

Unadjusted and adjusted analyses of potential predictors of SIP were performed (Table 2). After adjustment, immediate postpartum LARC placement was independently associated with a reduced risk of SIP (ARR: 0.28, 95% CI: 0.14-0.55, P < 0.001). Nulliparity, race, ethnicity, young maternal age, and marital status were not independent risk factors for SIP.

**Table 2:** Unadjusted analyses and adjusted models of predictors of short interpregnancy interval among patients who delivered between July 1, 2019, and March 31, 2020 Note: Bold numbers indicate statistically significant P-values. RR: risk ratio, CI: confidence interval, LARC: long-acting reversible contraception

Maternal characteristic	RR (95% CI)	P-value	Adjusted RR (95% CI)	P-value
Immediate postpartum LARC	0.33 (0.18-0.59)	<0.001	0.28 (0.14-0.55)	<0.001
First delivery	1.13 (0.90-1.41)	0.29	1.07 (0.76-1.50)	0.71
White race	1.08 (0.86-1.34)	0.52	0.96 (0.68-1.36)	0.84
Hispanic ethnicity	0.87 (0.69-1.09)	0.22	0.83 (0.58-1.17)	0.29
Maternal age range (18-21)	1.16 (0.87-1.54)	0.31	1.35 (0.86-2.11)	0.19
Marital status, married	1.30 (1.00-1.69)	0.05	1.42 (0.99-2.03)	0.06

## Discussion

Within the first nine months of the implementation of the postpartum LARC program at our health network, 12.4% of patients with state insurance opted for this method of contraception. This rate is slightly below the national average of 15.3% postpartum LARC use [8] and the Pennsylvania rate of 16.6% [9]. The CDC's Pregnancy Risk Assessment Monitoring System (PRAMS) reports a wider average range of 11.2%-37.5%, depending upon geographic region [8]. The lower rate seen in our sample may be attributable to the limited study time period and the recent introduction of the program.

Similar to a 2019 study by Oduyebo et al. [9], we observed a higher prevalence of LARC usage among younger patients and patients of color. Other studies have shown associations between race and/or ethnicity and a higher risk of SIP [10]. However, our study did not confirm those findings.

The overall SIP rate found in our population (246/1,126, 21.8%) matches up with findings from other studies focusing on patients with Medicaid coverage. Caldwell et al. reported SIP rates ranging from 21.2% to 23.9% [11], while Rodriguez et al. saw SIP rates of 15.2% to 42.1% within their study populations [12]. Rodriguez et al. also evaluated the timing and method of LARC for preventing SIP and concluded that any form of long-acting contraception was more effective than short-term pregnancy prevention methods (15.2%-23.0% SIP among patients opting for LARC versus 42.1% among those who used other contraceptive methods) [12].

Our study adds to the evidence supporting immediate postpartum LARC in patients interested in contraception after delivery. Providing access to LARC in the immediate postpartum period may reach those who might subsequently not have their contraceptive needs met [13]. Our study findings suggest that LARC access has the potential to reduce SIP rates within a healthcare organization's patient population. To this point, a longitudinal study by Champion et al. showed a significant increase in LARC uptake during its study period and an associated statistically significant increase in interpregnancy interval [14].

Our study had some limitations. We included only patients on public insurance, so we cannot generalize outcomes to those who are privately insured. We also did not study whether subsequent pregnancies among our patients were intentional. However, when we excluded the 732 patients who declined contraception or deferred the decision to the postpartum visit, the SIP rate remained low. Future research could expand our inquiry to include private insurance sets, evaluate maternal and infant outcomes of patients who experienced SIPs, and quantify whether subsequent pregnancies had been planned. Other potential research directions include following up with patients who declined immediate postpartum LARC to determine whether they ultimately initiated LARC or other contraceptive methods or a comparative effectiveness study between immediate postpartum LARC and other contraceptive methods in preventing SIP.

## Conclusions

The program at our health network to offer immediate postpartum LARC to patients shows initial promise for uptake and prevention of short interpregnancy intervals. Larger studies that include privately insured patients and determine pregnancy intention are warranted. Encouraging patients interested in long-term contraception to consider immediate postpartum LARC is one way clinicians can support patients in their family planning decisions.
